# Binding constants of *Southern rice black-streaked dwarf virus* Coat Protein with ferulic acid derivatives

**DOI:** 10.1016/j.dib.2018.01.031

**Published:** 2018-01-31

**Authors:** Longlu Ran, Yan Ding, Liangzhi Luo, Xiuhai Gan, Xiangyang Li, Yongzhong Chen, Deyu Hu, Baoan Song

**Affiliations:** State Key Laboratory Breeding Base of Green Pesticide and Agricultural Bioengineering, Key Laboratory of Green Pesticide and Agricultural Bioengineering, Ministry of Education, Guizhou University, Huaxi District, Guiyang 550025, PR China

## Abstract

The data present binding constants between ferulic acid derivatives and the Coat Protein (P10) by fluorescence titration in this article, which is hosted in the research article entitled “Interaction Research on an Antiviral Molecule that Targets the Coat Protein of *Southern rice black-streaked dwarf virus*’’ (Ran et al., 2017) [1]. The data include fluorescence quenching spectrum, Stern–Volmer quenching constants, and binding parameters. In this article, a more comprehensive data interpretation and analysis is explained.

**Specifications Table**

**Table:** 

Subject area	*Biology*
More specific subject area	Biochemistry, Protein and drug interactions
Type of data	Figure
How data was acquired	The Fluorescence quenching spectra were acquired by FluoroMax®-4P fluorescence spectrophotometer.
Data format	Analyzed
Experimental factors	Coat Protein (P10) was purified from *Escherichia coli*. Ferulic acid derivatives were synthesized in our previous work.
Experimental features	P10 concentration was fixed and the concentration of the ferulic acid derivative gradually increased, and then the fluorescence intensity of the mixture was recorded in the wavelength range of 290–450 nm.
Data source location	*Guiyang, China*
Data accessibility	The data are included with this article

**Value of the data**

• The data serve as a background for the affinity of P10 with ferulic acid derivatives by fluorescence titration.
• The data present a method for interaction research of molecule and target protein.
• The data provide a new insight for further design novel and potent anti-*Southern rice black-streaked dwarf virus* compounds.

## 1 Data

**Fig. 1:**
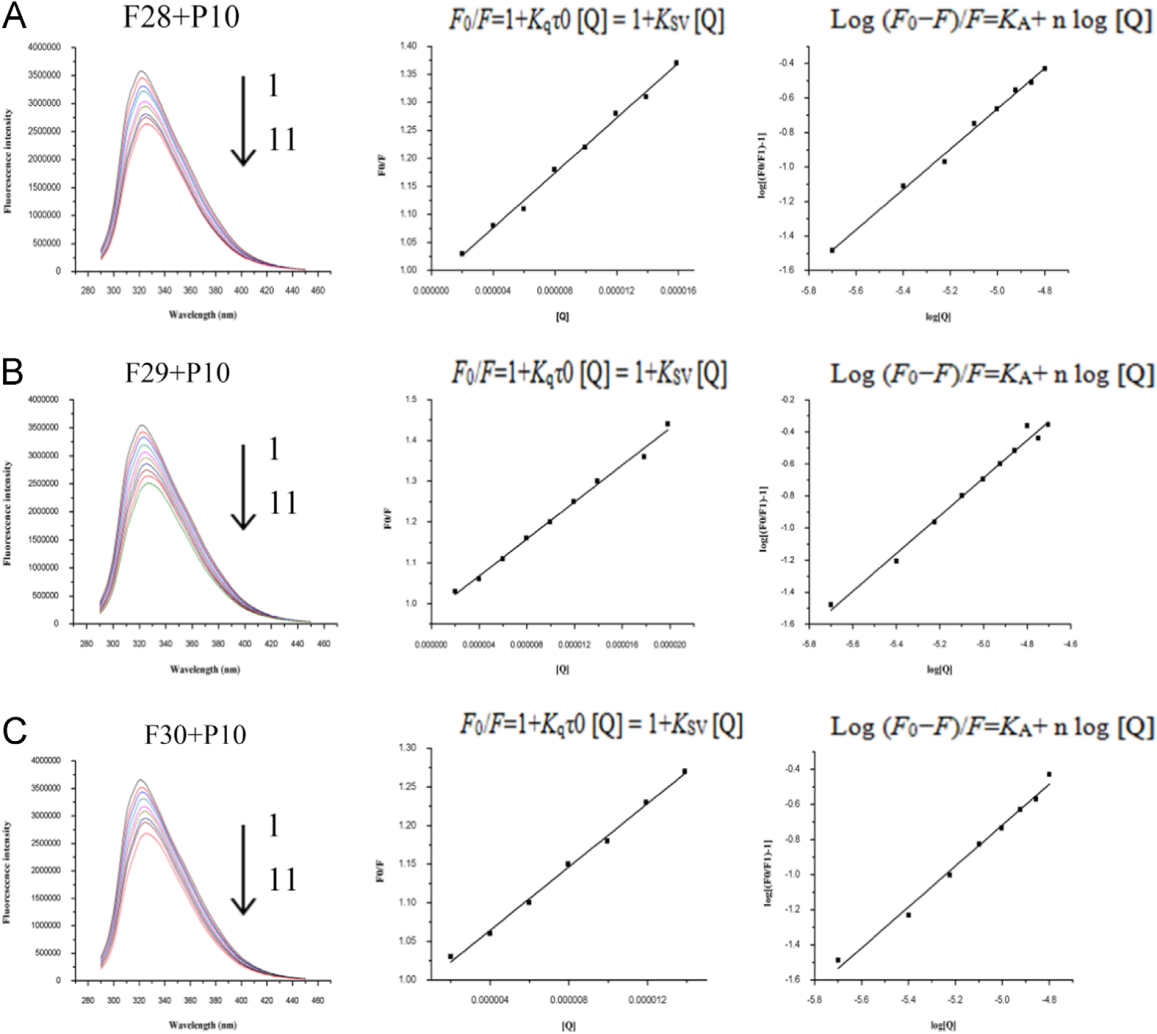
Fluorescence quenching spectra of P10 in the presence of compounds F28, F29, F30. (A) Concentration of P10 was 5 μM; (1–11) the drug concentrations of F28 was 0, 2.0, 4.0, 6.0, 8.0, 10.0, 12.0, 14.0, 16.0, 18.0, and 20.0 μM; (B) Concentration of P10 was 5 μM; (1–11) the drug concentrations of F29 was 0, 2.0, 4.0, 6.0, 8.0, 10.0, 12.0, 14.0, 16.0, 18.0, and 20.0 μM; (C) Concentration of P10 was 5 μM; (1–11) the drug concentrations of F30 was 0, 2.0, 4.0, 6.0, 8.0, 10.0, 12.0, 14.0, 16.0, 18.0, and 20.0 μM.

The dataset of this article provide information on the interaction of 33 ferulic acid derivatives with P10 protein by fluorescence titration. The fluorescence quenching spectrum for compounds F28, F29, F30 are shown in Fig. 1, and the other compounds are shown in Supplementary data 1.

## 2 Experimental design, materials and methods

P10 protein was purified from *Escherichia coli* [1]. All the compounds were dissolved in buffer (20 mM Tris and 150 mM NaCl, pH 8.0) at μΜ concentrations [containing 5% N, N-dimethylformamide (DMF) (v/v)].

At 275 nm excitation wavelength, the fluorescence quenching spectra were recorded at 298 K from 290 nm to 450 nm when the excitation and emission band slit widths were both 5 nm. Fluorescence quenching can be dynamic or static. Such type of fluorescence quenching can be verified through the Stern–Volmer equation, as shown in Eq. (1) [2], [3].

(1) $$F_{0}/F=1+K_{q}\tau 0\left[Q\right]=1+K_{SV}\left[Q\right]$$

The maximum quenching constant of the quencher on the biomacromolecule is about 2×10^10^ M^−1^ S^−1^. When *K*q > 2×10^10^ M^−1^ S^−1^, the main reason for the fluorescence quenching is static quenching [4]. The binding constants *K*_A_ and binding sites n of the complex can be calculated using Eq. (2) [5].

(2) $$Log\left(F_{0}-F\right)/F=K_{A}+nlog\left[Q\right]$$

## Appendix A Supplementary material

Transparency document
