# Supplementary material for: Binding constants of Southern rice black-streaked dwarf virus Coat Protein with ferulic acid derivatives
Source: Data Brief. 2018 Jan 31;17:321–4. doi: 10.1016/j.dib.2018.01.031 (PMC5988289; doi:10.1016/j.dib.2018.01.031)
Supplement: Supplementary file 2 — Transparency document [file mmc2.docx]

Fluorescence quenching spectra of P10 in the presence of ferulic acid derivatives


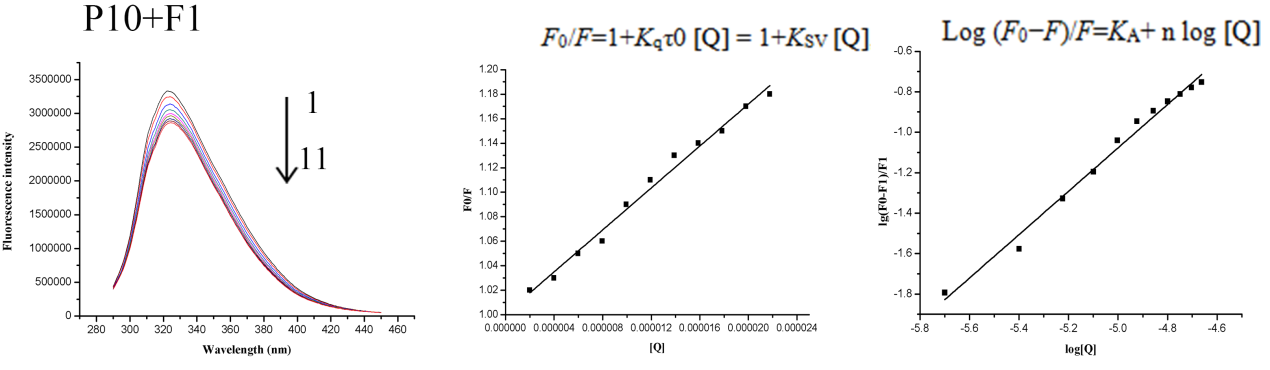


**Fig.S1** Fluorescence quenching spectra of P10 in the presence of F1.

(1) Free P10 (5 μM); (1–11) the drug concentrations of F1 was 0, 2.0, 4.0, 6.0, 8.0, 10.0, 12.0, 14.0, 16.0, 18.0, and 20.0 μM.


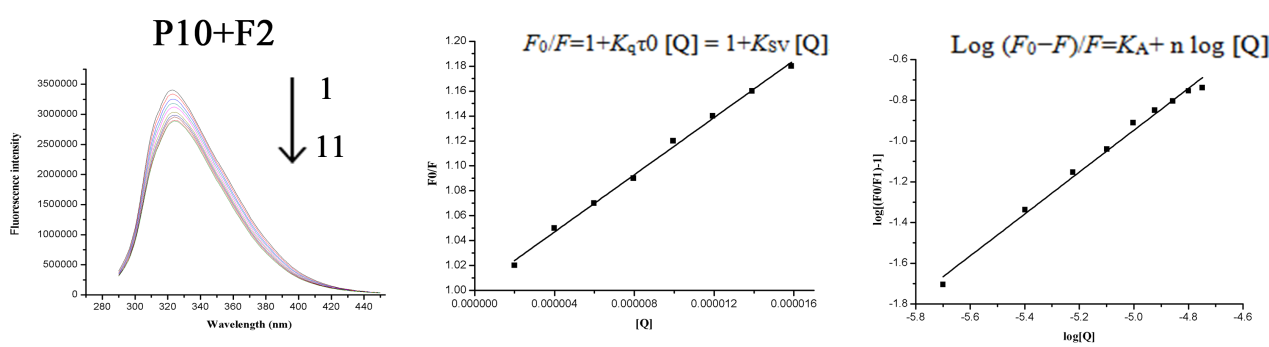


**Fig.S2** Fluorescence quenching spectra of P10 in the presence of F2.

(1) Free P10 (5 μM); (1–11) the drug concentrations of F2 was 0, 2.0, 4.0, 6.0, 8.0, 10.0, 12.0, 14.0, 16.0, 18.0, and 20.0 μM.


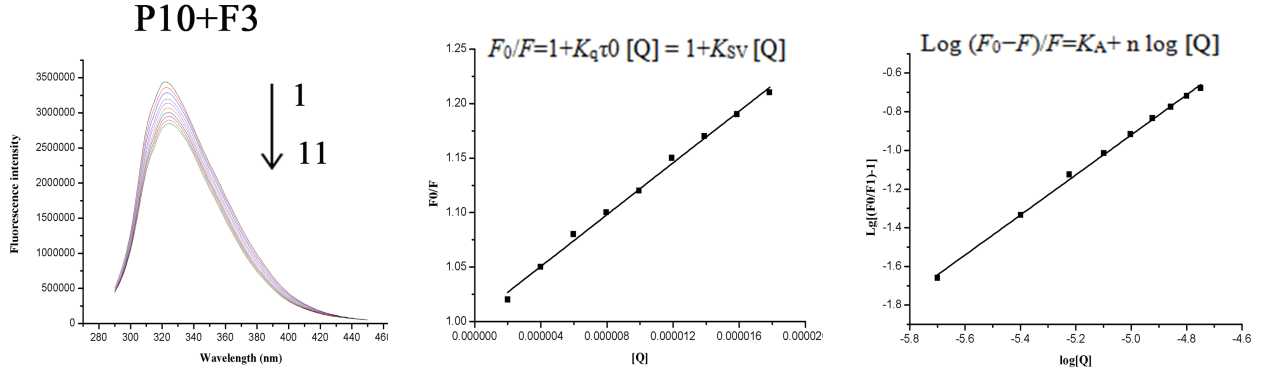


**Fig.S3** Fluorescence quenching spectra of P10 in the presence of F3.

(1) Free P10 (5 μM); (1–11) the drug concentrations of F3 was 0, 2.0, 4.0, 6.0, 8.0, 10.0, 12.0, 14.0, 16.0, 18.0, and 20.0 μM.


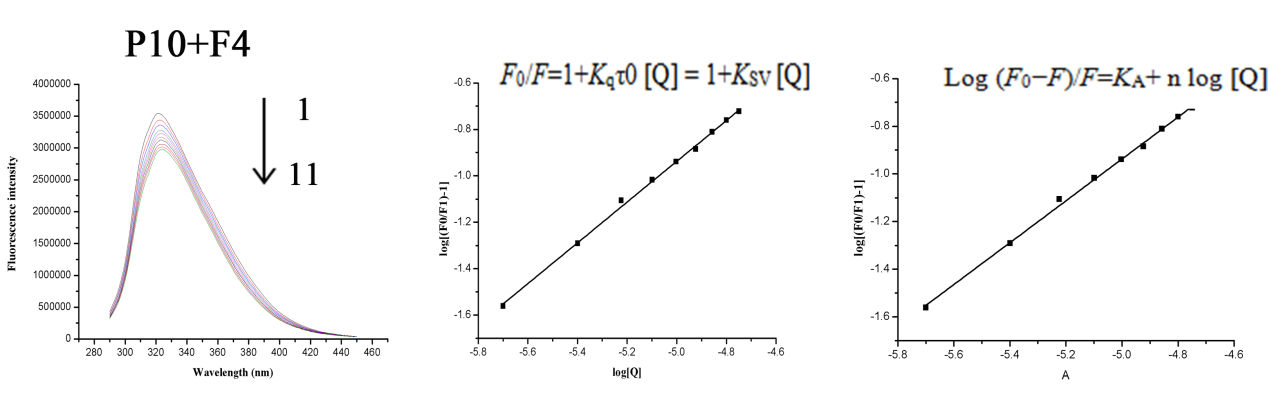


**Fig.S4** Fluorescence quenching spectra of P10 in the presence of F4.

(1) Free P10 (5 μM); (1–11) the drug concentrations of F4 was 0, 2.0, 4.0, 6.0, 8.0, 10.0, 12.0, 14.0, 16.0, 18.0, and 20.0 μM.


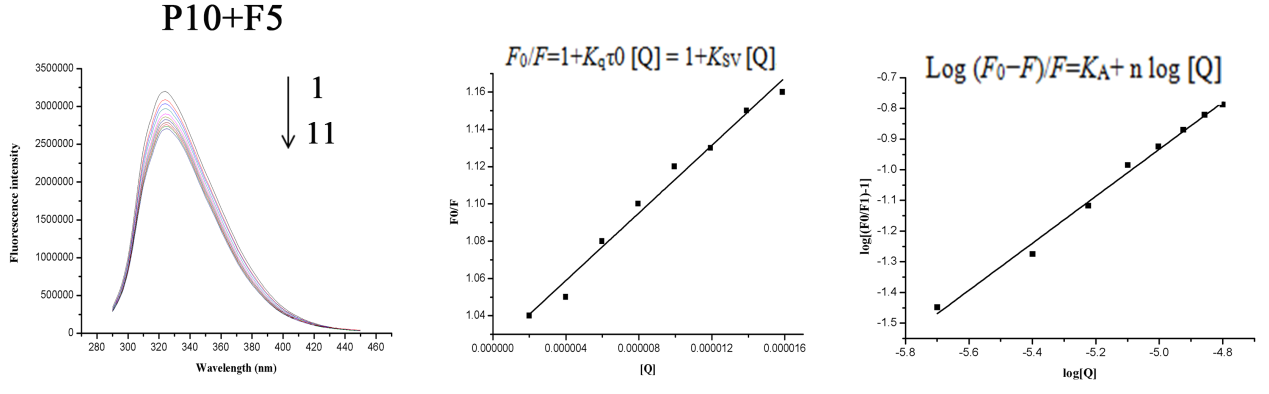


**Fig.S5** Fluorescence quenching spectra of P10 in the presence of F5.

(1) Free P10 (5 μM); (1–11) the drug concentrations of F5 was 0, 2.0, 4.0, 6.0, 8.0, 10.0, 12.0, 14.0, 16.0, 18.0, and 20.0 μM.


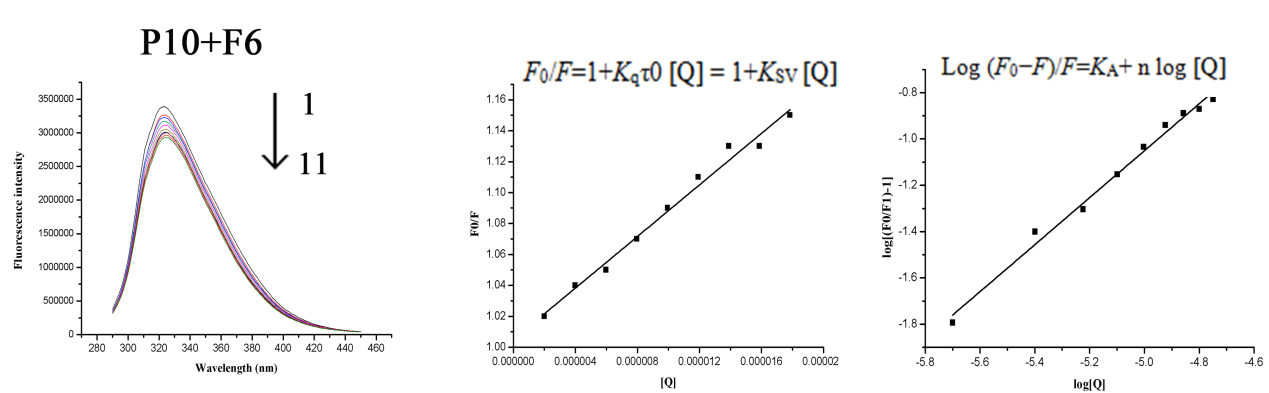


**Fig.S6** Fluorescence quenching spectra of P10 in the presence of F6.

(1) Free P10 (5 μM); (1–11) the drug concentrations of F6 was 0, 2.0, 4.0, 6.0, 8.0, 10.0, 12.0, 14.0, 16.0, 18.0, and 20.0 μM.


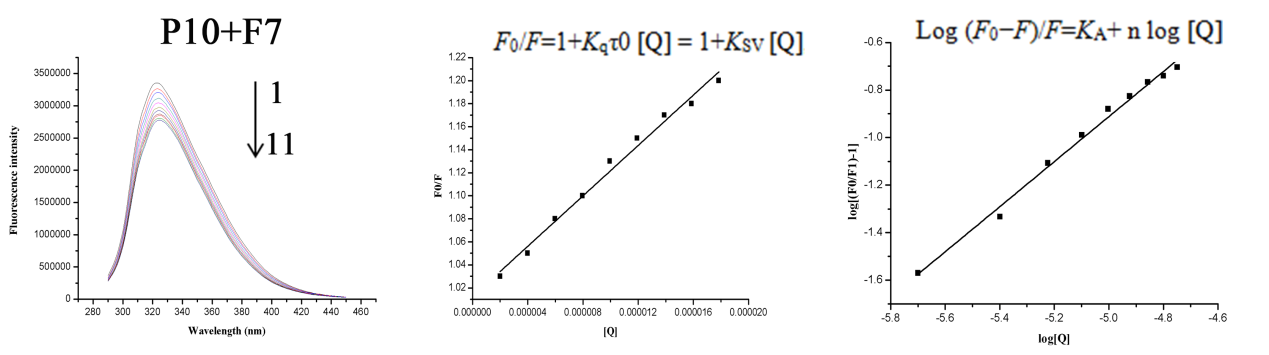


**Fig.S7** Fluorescence quenching spectra of P10 in the presence of F7.

(1) Free P10 (5 μM); (1–11) the drug concentrations of F7 was 0, 2.0, 4.0, 6.0, 8.0, 10.0, 12.0, 14.0, 16.0, 18.0, and 20.0 μM.


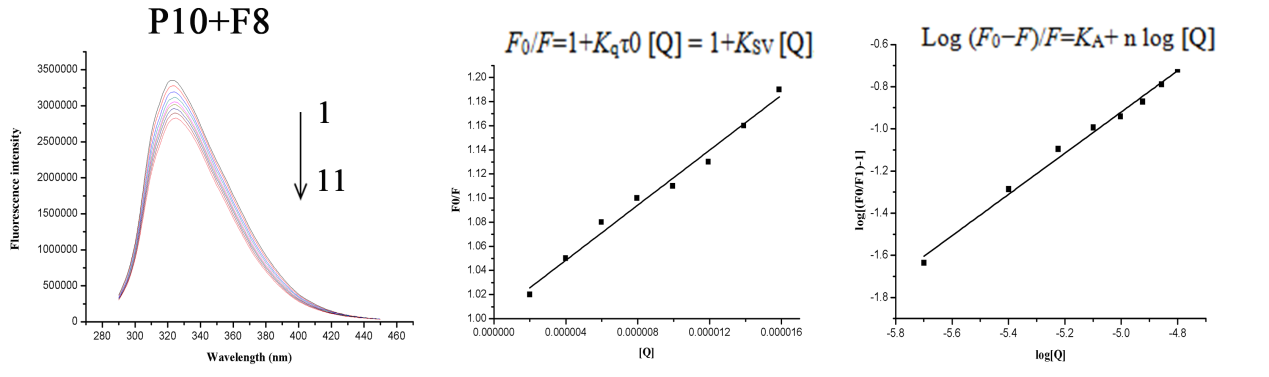


**Fig.S8** Fluorescence quenching spectra of P10 in the presence of F8.

(1) Free P10 (5 μM); (1–11) the drug concentrations of F8 was 0, 2.0, 4.0, 6.0, 8.0, 10.0, 12.0, 14.0, 16.0, 18.0, and 20.0 μM.


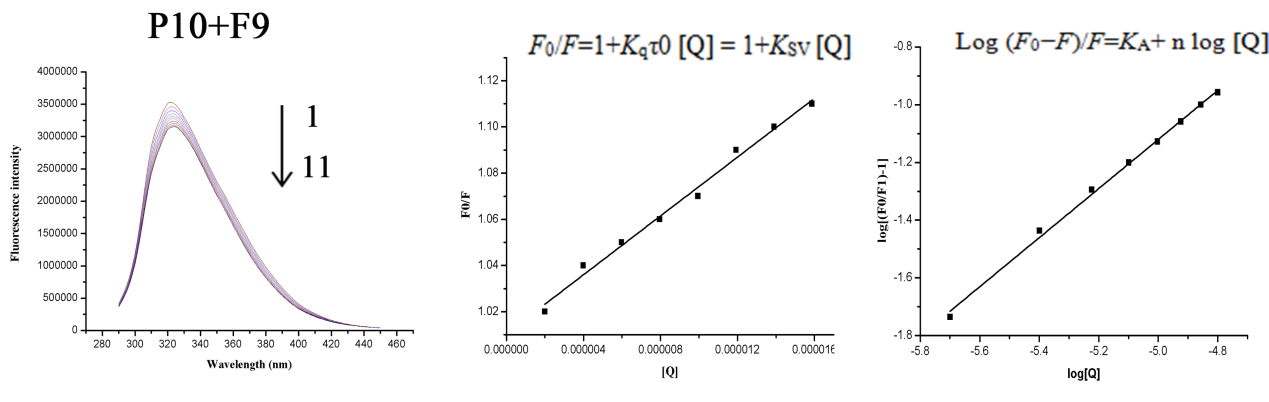


**Fig.S9** Fluorescence quenching spectra of P10 in the presence of F9.

(1) Free P10 (5 μM); (1–11) the drug concentrations of F9 was 0, 2.0, 4.0, 6.0, 8.0, 10.0, 12.0, 14.0, 16.0, 18.0, and 20.0 μM.


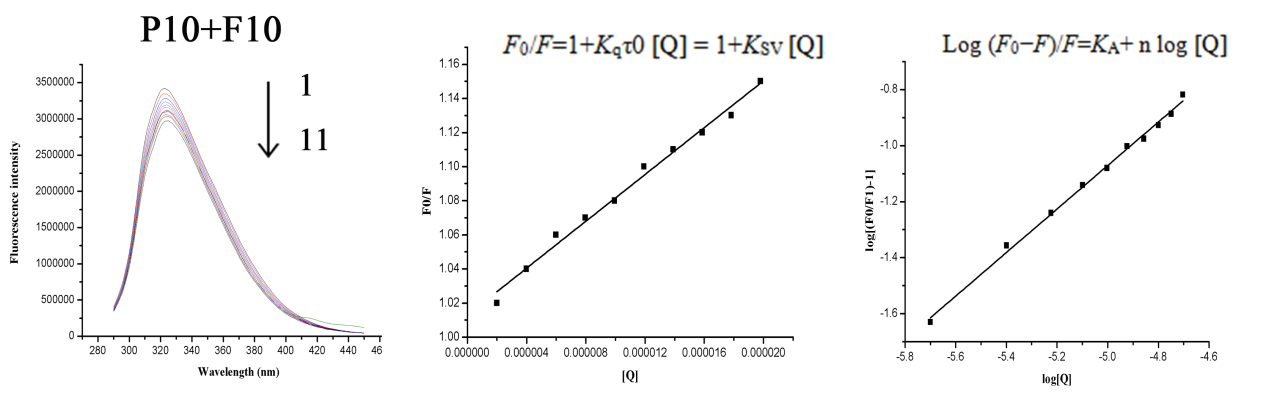


**Fig.S10** Fluorescence quenching spectra of P10 in the presence of F10.

(1) Free P10 (5 μM); (1–11) the drug concentrations of F10 was 0, 2.0, 4.0, 6.0, 8.0, 10.0, 12.0, 14.0, 16.0, 18.0, and 20.0 μM.


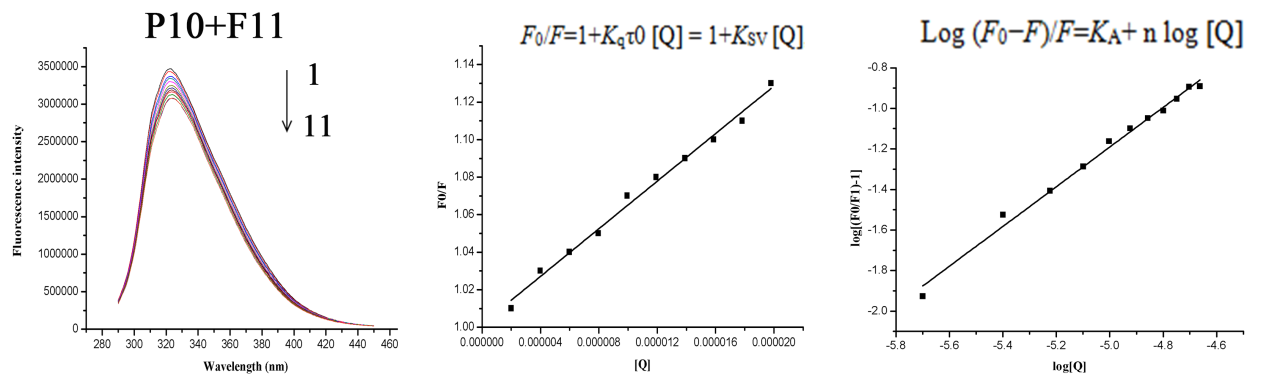


**Fig.S11** Fluorescence quenching spectra of P10 in the presence of F11.

(1) Free P10 (5 μM); (1–11) the drug concentrations of F1 was 0, 2.0, 4.0, 6.0, 8.0, 10.0, 12.0, 14.0, 16.0, 18.0, and 20.0 μM.


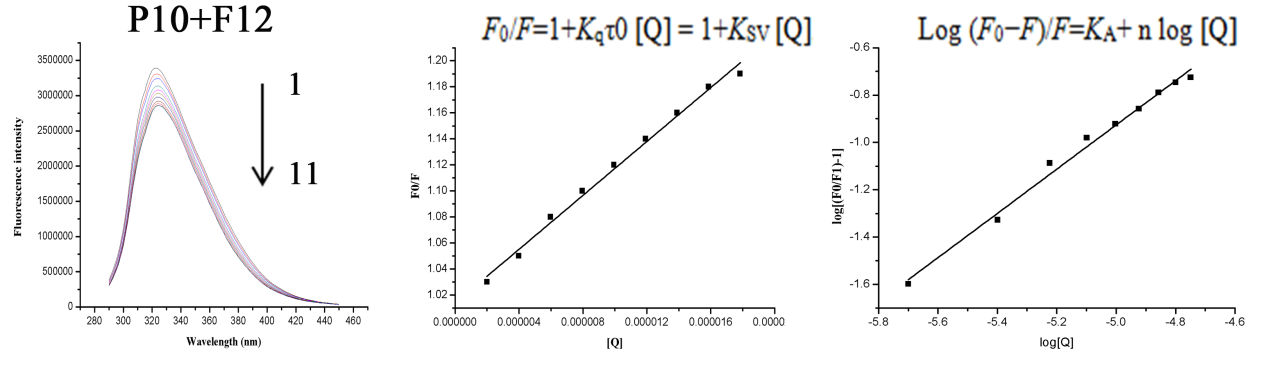


**Fig.S12** Fluorescence quenching spectra of P10 in the presence of F12.

(1) Free P10 (5 μM); (1–11) the drug concentrations of F12 was 0, 2.0, 4.0, 6.0, 8.0, 10.0, 12.0, 14.0, 16.0, 18.0, and 20.0 μM.


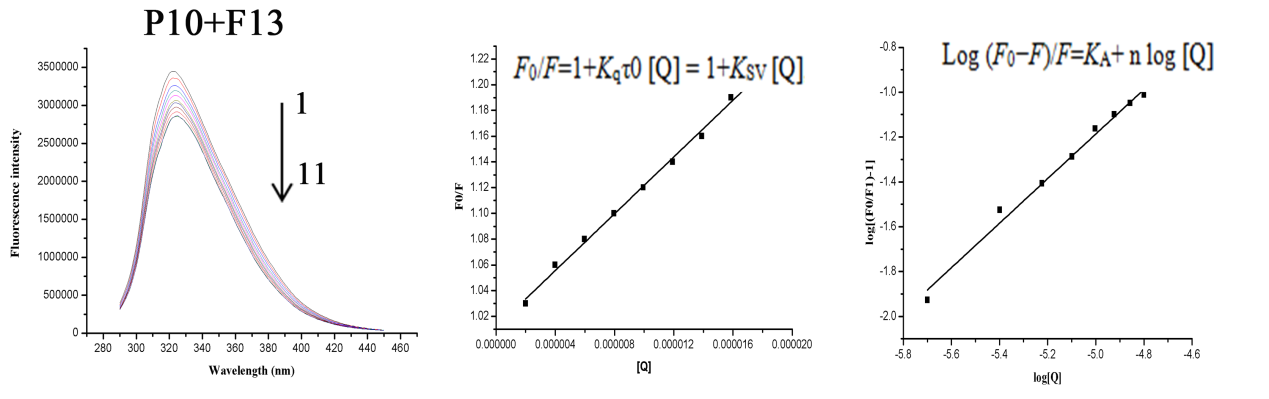


**Fig.S13** Fluorescence quenching spectra of P10 in the presence of F13.

(1) Free P10 (5 μM); (1–11) the drug concentrations of F13 was 0, 2.0, 4.0, 6.0, 8.0, 10.0, 12.0, 14.0, 16.0, 18.0, and 20.0 μM.


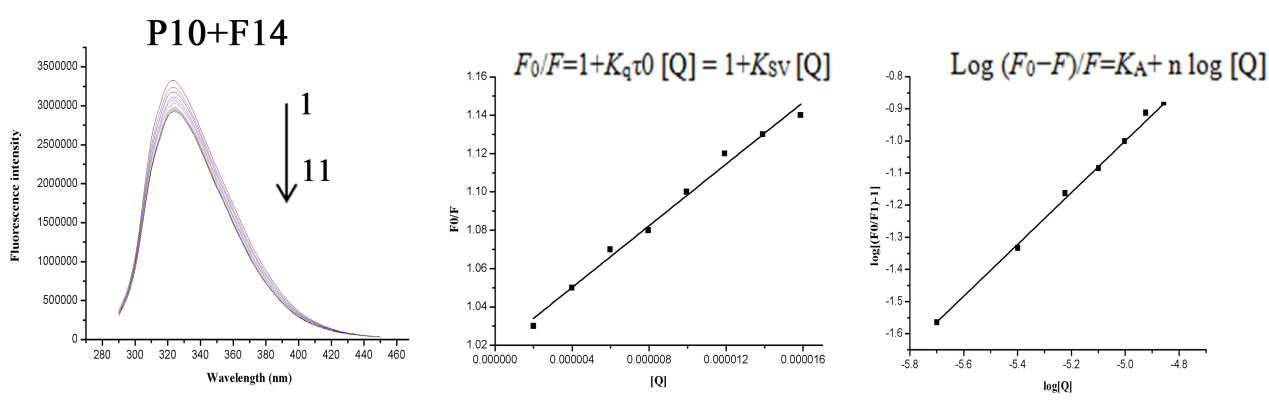


**Fig.S14** Fluorescence quenching spectra of P10 in the presence of F14.

(1) Free P10 (5 μM); (1–11) the drug concentrations of F14 was 0, 2.0, 4.0, 6.0, 8.0, 10.0, 12.0, 14.0, 16.0, 18.0, and 20.0 μM.


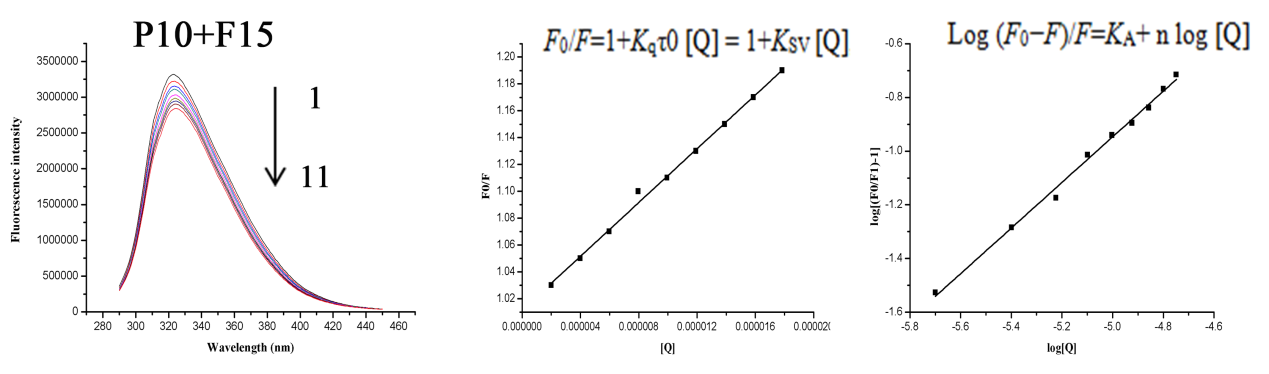


**Fig.S15** Fluorescence quenching spectra of P10 in the presence of F15.

(1) Free P10 (5 μM); (1–11) the drug concentrations of F15 was 0, 2.0, 4.0, 6.0, 8.0, 10.0, 12.0, 14.0, 16.0, 18.0, and 20.0 μM.


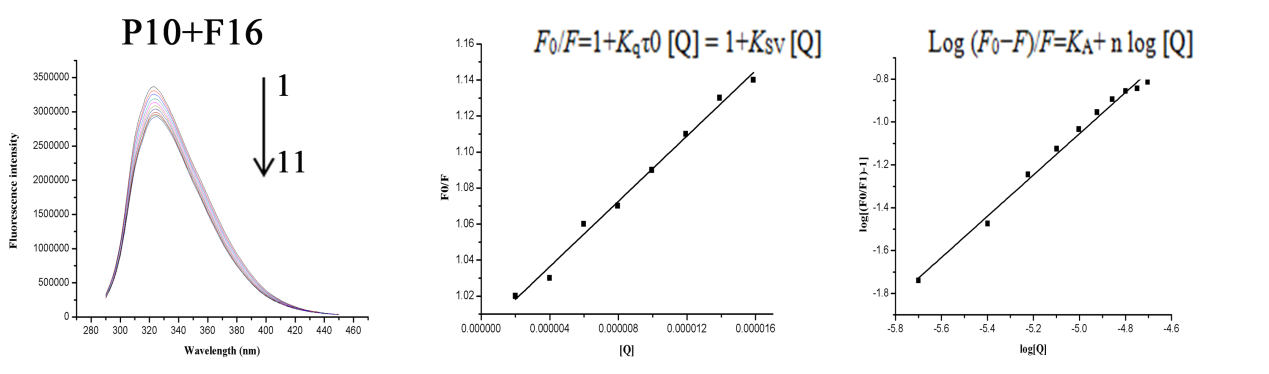


**Fig.S16** Fluorescence quenching spectra of P10 in the presence of F16.

(1) Free P10 (5 μM); (1–11) the drug concentrations of F16 was 0, 2.0, 4.0, 6.0, 8.0, 10.0, 12.0, 14.0, 16.0, 18.0, and 20.0 μM.


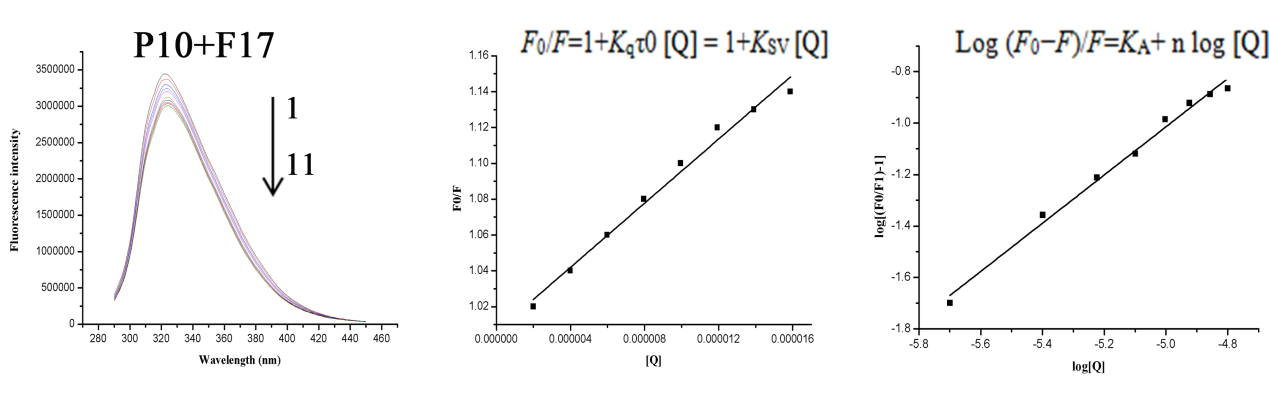


**Fig.S17** Fluorescence quenching spectra of P10 in the presence of F17.

(1) Free P10 (5 μM); (1–11) the drug concentrations of F17 was 0, 2.0, 4.0, 6.0, 8.0, 10.0, 12.0, 14.0, 16.0, 18.0, and 20.0 μM.


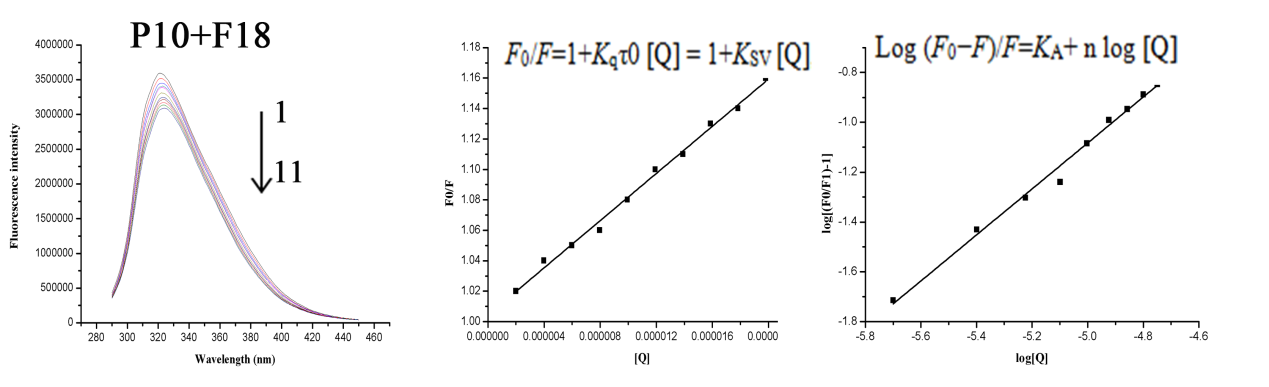


**Fig.S18** Fluorescence quenching spectra of P10 in the presence of F18.

(1) Free P10 (5 μM); (1–11) the drug concentrations of F18 was 0, 2.0, 4.0, 6.0, 8.0, 10.0, 12.0, 14.0, 16.0, 18.0, and 20.0 μM.


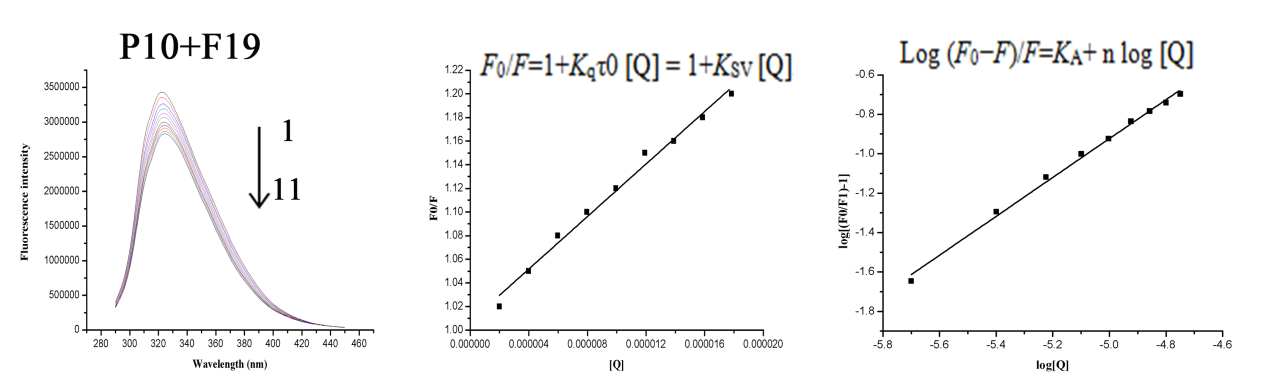


**Fig.S19** Fluorescence quenching spectra of P10 in the presence of F19.

(1) Free P10 (5 μM); (1–11) the drug concentrations of F19 was 0, 2.0, 4.0, 6.0, 8.0, 10.0, 12.0, 14.0, 16.0, 18.0, and 20.0 μM.


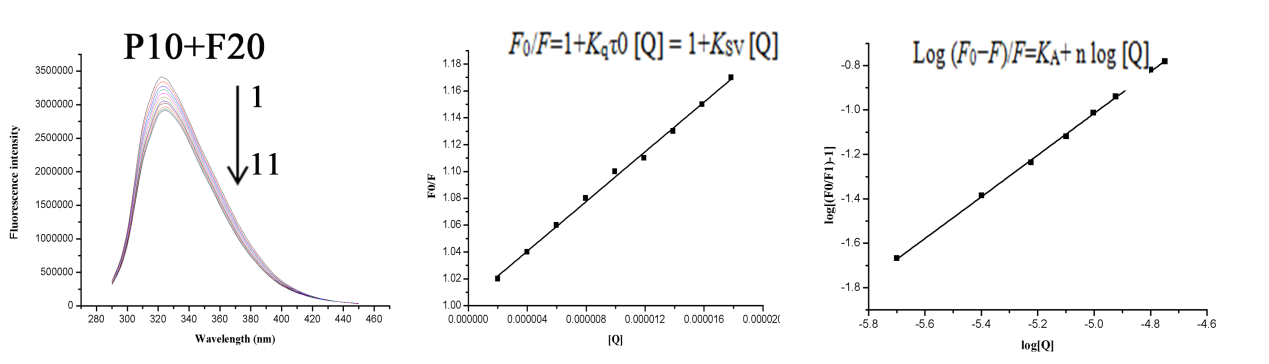


**Fig.S20** Fluorescence quenching spectra of P10 in the presence of F20.

(1) Free P10 (5 μM); (1–11) the drug concentrations of F20 was 0, 2.0, 4.0, 6.0, 8.0, 10.0, 12.0, 14.0, 16.0, 18.0, and 20.0 μM.


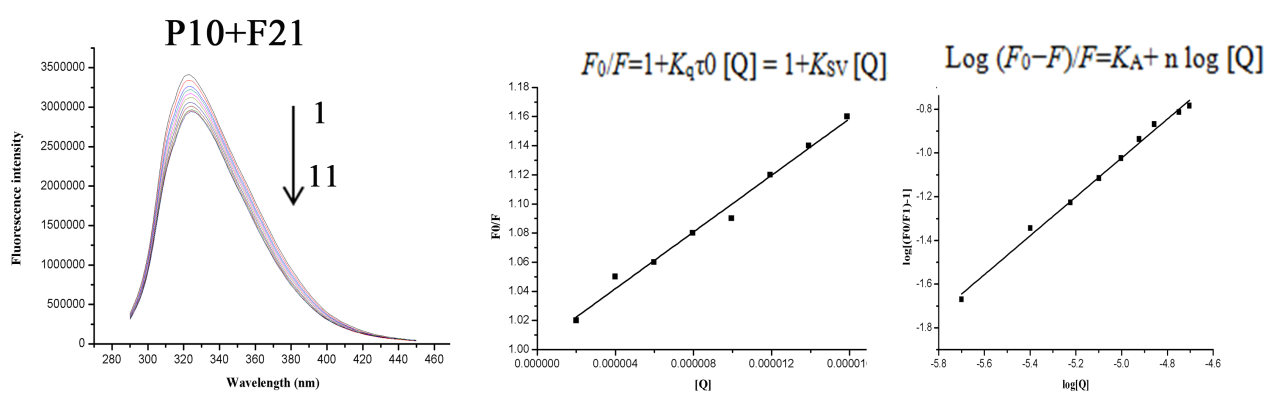


**Fig.S21** Fluorescence quenching spectra of P10 in the presence of F21.

(1) Free P10 (5 μM); (1–11) the drug concentrations of F21 was 0, 2.0, 4.0, 6.0, 8.0, 10.0, 12.0, 14.0, 16.0, 18.0, and 20.0 μM.


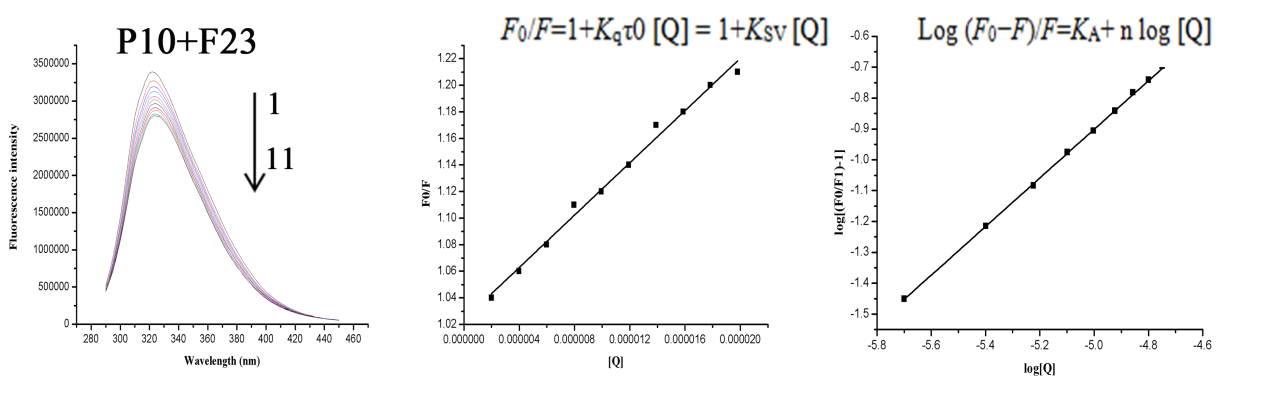


**Fig.S22** Fluorescence quenching spectra of P10 in the presence of F23.

(1) Free P10 (5 μM); (1–11) the drug concentrations of F23 was 0, 2.0, 4.0, 6.0, 8.0, 10.0, 12.0, 14.0, 16.0, 18.0, and 20.0 μM.


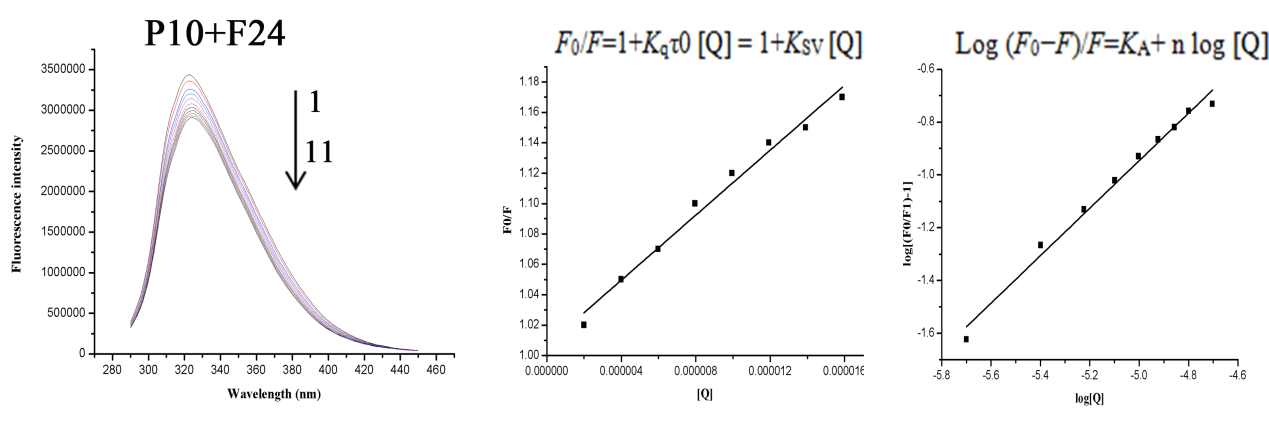


**Fig.S23** Fluorescence quenching spectra of P10 in the presence of F24.

(1) Free P10 (5 μM); (1–11) the drug concentrations of F24 was 0, 2.0, 4.0, 6.0, 8.0, 10.0, 12.0, 14.0, 16.0, 18.0, and 20.0 μM.


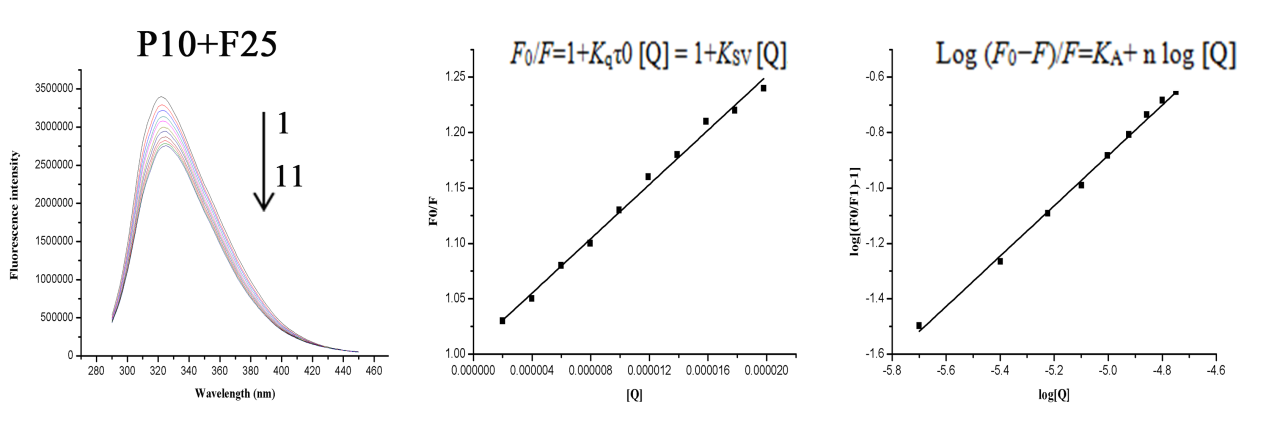


**Fig.S24** Fluorescence quenching spectra of P10 in the presence of F25.

(1) Free P10 (5 μM); (1–11) the drug concentrations of F25 was 0, 2.0, 4.0, 6.0, 8.0, 10.0, 12.0, 14.0, 16.0, 18.0, and 20.0 μM.


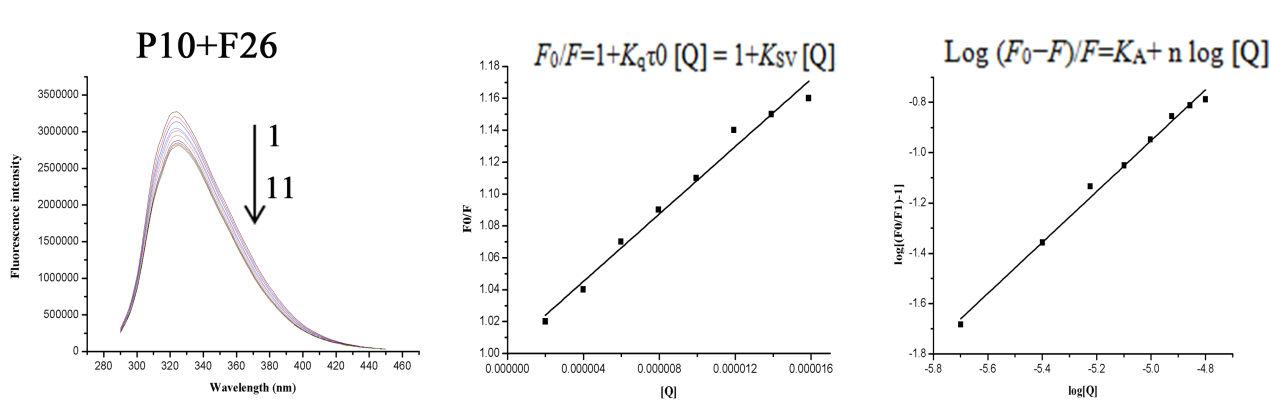


**Fig.S25** Fluorescence quenching spectra of P10 in the presence of F26.

(1) Free P10 (5 μM); (1–11) the drug concentrations of F26 was 0, 2.0, 4.0, 6.0, 8.0, 10.0, 12.0, 14.0, 16.0, 18.0, and 20.0 μM.


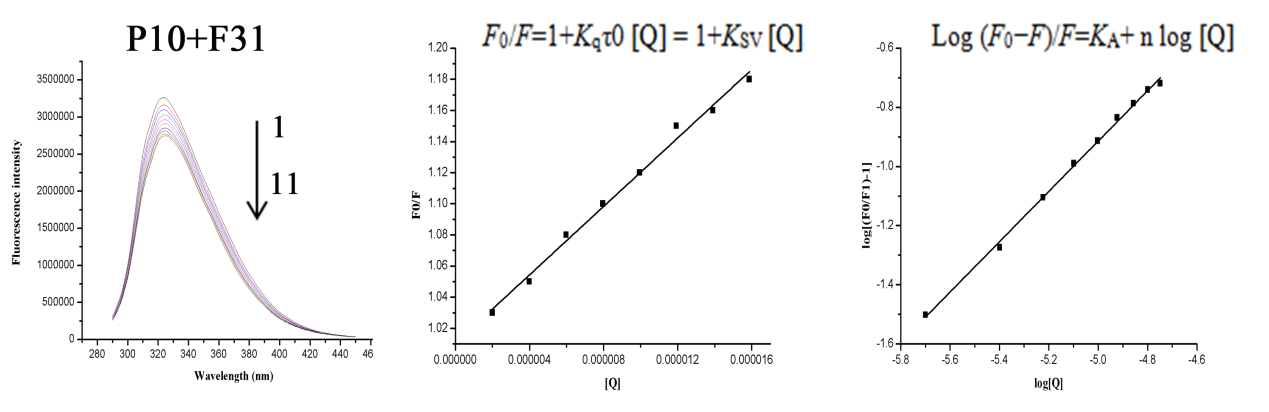


**Fig.S26** Fluorescence quenching spectra of P10 in the presence of F31.

(1) Free P10 (5 μM); (1–11) the drug concentrations of F31 was 0, 2.0, 4.0, 6.0, 8.0, 10.0, 12.0, 14.0, 16.0, 18.0, and 20.0 μM.


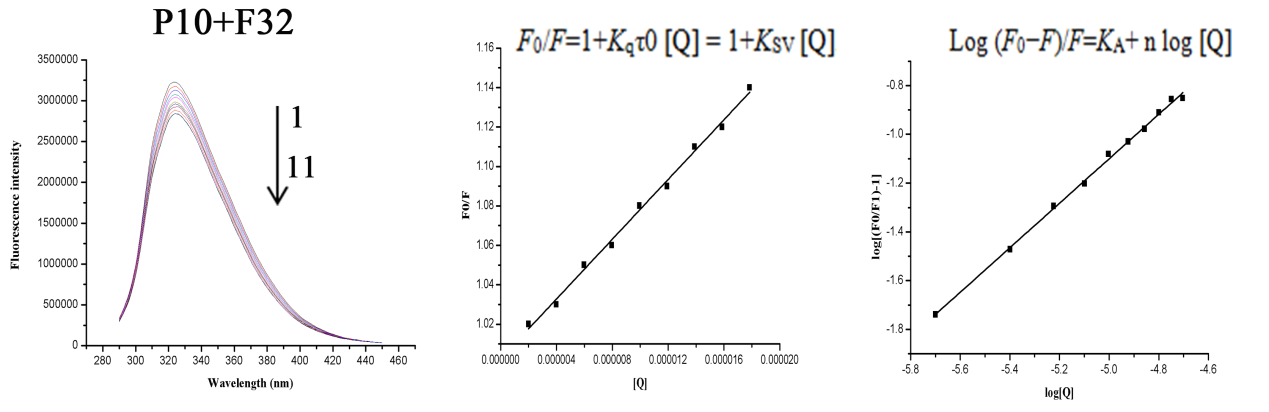


**Fig.S27** Fluorescence quenching spectra of P10 in the presence of F32.

(1) Free P10 (5 μM); (1–11) the drug concentrations of F32 was 0, 2.0, 4.0, 6.0, 8.0, 10.0, 12.0, 14.0, 16.0, 18.0, and 20.0 μM.


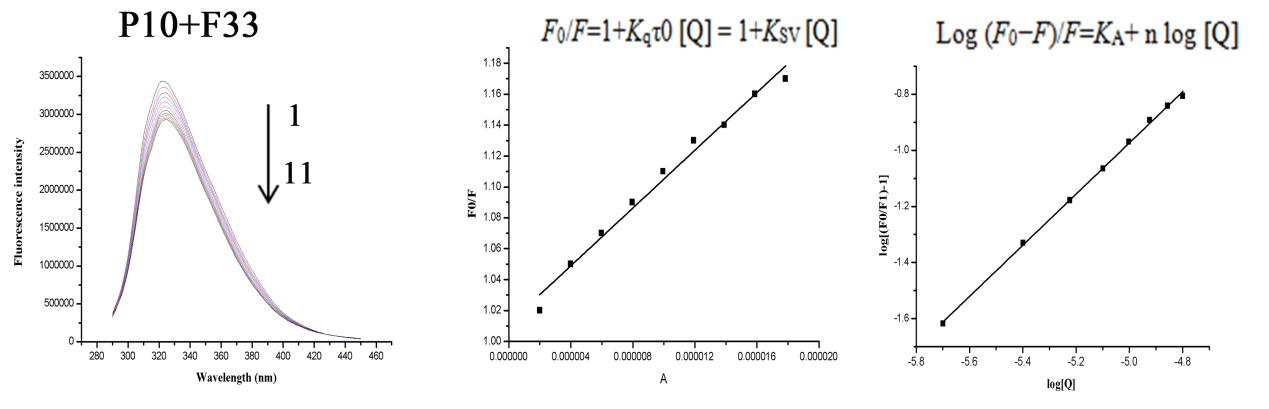


**Fig.S28** Fluorescence quenching spectra of P10 in the presence of F33.

(1) Free P10 (5 μM); (1–11) the drug concentrations of F33 was 0, 2.0, 4.0, 6.0, 8.0, 10.0, 12.0, 14.0, 16.0, 18.0, and 20.0 μM.


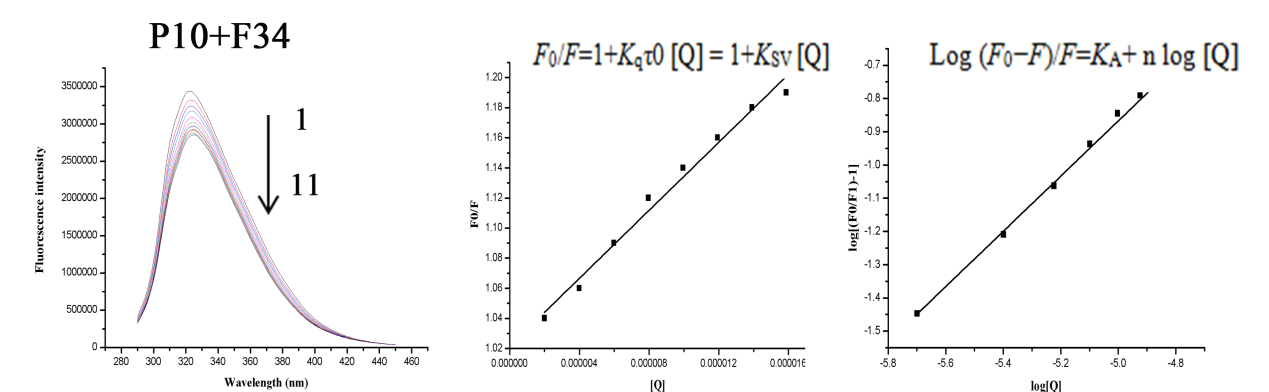


**Fig.S29** Fluorescence quenching spectra of P10 in the presence of F34.

(1) Free P10 (5 μM); (1–11) the drug concentrations of F34 was 0, 2.0, 4.0, 6.0, 8.0, 10.0, 12.0, 14.0, 16.0, 18.0, and 20.0 μM.


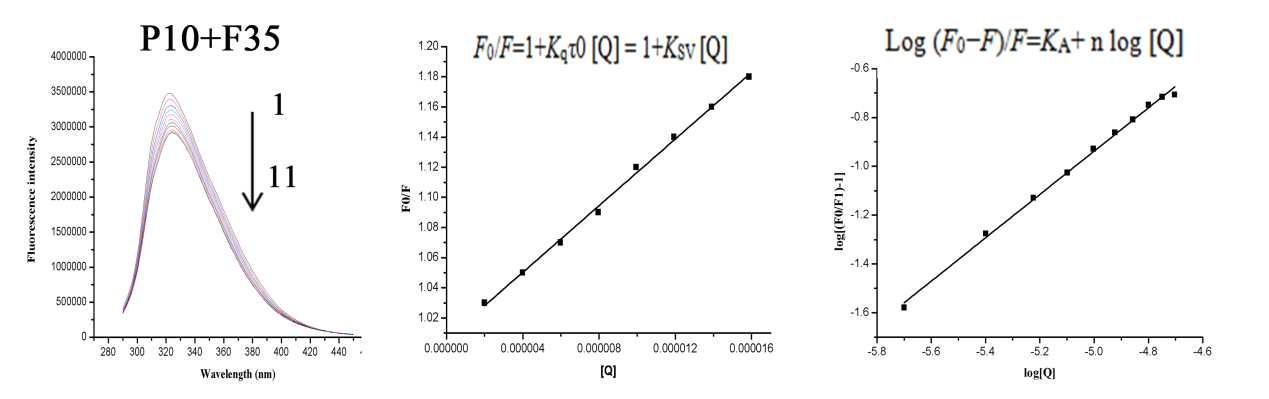


**Fig.S30** Fluorescence quenching spectra of P10 in the presence of F35.

(1) Free P10 (5 μM); (1–11) the drug concentrations of F35 was 0, 2.0, 4.0, 6.0, 8.0, 10.0, 12.0, 14.0, 16.0, 18.0, and 20.0 μM.
